# Proteomic Profile of Saliva in Parkinson’s Disease Patients: A Proof of Concept Study

**DOI:** 10.3390/brainsci11050661

**Published:** 2021-05-18

**Authors:** Monika Figura, Ewa Sitkiewicz, Bianka Świderska, Łukasz Milanowski, Stanisław Szlufik, Dariusz Koziorowski, Andrzej Friedman

**Affiliations:** 1 Department of Neurology, Faculty of Health Sciences, Medical University of Warsaw, 03-242 Warsaw, Poland; lukasz.milanowski91@gmail.com (Ł.M.); stanislaw.szlufik@wum.edu.pl (S.S.); dariusz.koziorowski@wum.edu.pl (D.K.); andrzej.friedman@wum.edu.pl (A.F.); 2 Mass Spectrometry Laboratory, Institute of Biochemistry and Biophysics Polish Academy of Sciences, 02-106 Warsaw, Poland; ewa@ibb.waw.pl (E.S.); bianka.swiderska@gmail.com (B.Ś.)

**Keywords:** Parkinson’s disease, saliva, proteomic profile, neurodegeneration, salivary glands

## Abstract

Parkinson’s disease (PD) is a progressive neurodegenerative disorder. It affects many organs. Lewy bodies—a histopathological “hallmark” of PD—are detected in about 75% of PD submandibular gland samples. We hypothesize that saliva can be a source of biomarkers of PD. The aim of the study was to evaluate and compare the salivary proteome of PD patients and healthy controls (HC). Salivary samples from 39 subjects (24 PD patients, mean age 61.6 ± 8.2; 15 HC, mean age 60.9 ± 6.7) were collected. Saliva was collected using RNA-Pro-Sal kits. Label-free LC-MS/MS mass spectrometry was performed to characterize the proteome of the saliva. IPA analysis of upstream inhibitors was performed. A total of 530 proteins and peptides were identified. We observed lower concentrations of S100-A16, ARP2/3, and VPS4B in PD group when compared to HC. We conclude that the salivary proteome composition of PD patients is different than that of healthy controls. We observed a lower concentration of proteins involved in inflammatory processes, exosome formation, and adipose tissue formation. The variability of expression of proteins between the two groups needs to be considered.

## 1. Introduction

Despite being the second most frequent neurodegenerative disorder, Parkinson’s disease (PD) is still diagnosed in its late motor stage. The diagnosis becomes possible only when the pathology has spread throughout the nervous system, causing damage to over 50% of the substantia nigra cells [1]. The only route to early intervention in PD treatment is through the development of biomarkers. The current availability of biomarkers facilitating early PD diagnosis is limited. Diagnosis is established with limited accuracy on the basis of clinical symptoms of bradykinesia with concomitant muscular rigidity or rest tremor or postural instability [2]. Diagnostic tools such as a DAT scan, transcranial ultrasonography of the substantia nigra, olfactory test, or autonomic assessment may be of help, but due to high costs and limited accessibility they cannot be used for routine screening of all suspected cases.

Looking for biomarkers of PD-related degeneration seems to be the crucial next step in its early diagnosis. Current efforts such as the BIOFIND initiative or Systemic Synuclein Sampling Study are directed towards the identification of tissue that may serve as a biomarker source [3,4]. Organs such as salivary glands, gastroenteric plexuses, adrenal glands, or the urinary system seem to be involved in PD pathology long before motor symptoms develop [5,6,7,8]. They are also more feasible for sample collection than the CNS nervous tissue. Staining for alpha-synuclein deposits (Lewy bodies and neurites) performed in samples from peripheral tissues is currently being investigated as a biomarker of PD. Protein misfolding cyclic amplification (PMCA) and real-time quaking-induced conversion (RT-QuIC) techniques seem to be promising, new, ultrasensitive tools for the detection of very small amounts of alpha-synuclein prone to forming aggregates [9].

Saliva production is abnormal in PD, with higher total protein concentrations [10]. In addition to drooling, which is commonly associated with PD, xerostomia is also frequent among PD patients, being reported in 60.8% of cases [11]. Detailed findings on salivary protein changes in PD were summarized in a paper by our group [12]. We concluded that higher concentrations of oligomeric alpha-synuclein seem to be the most promising salivary biomarker of PD [13,14]. Oligomeric alpha-synuclein seems to be a key player in PD-related neurodegeneration. It is involved in mitochondrial dysfunction and inefficient removal of misfolded proteins by proteasomes, synaptic dysfunction, and neuroinflammation, among others [15,16,17]. Its higher expression in saliva supports the idea that the neurodegenerative process in PD is generalized and may be reflected in saliva composition.

Human saliva has several functions. It contains many proteins and peptides as its components. Its organic components include amylases, cystatins, hormones, lysozyme, lipase, lactoferrins, mucins, peroxidase, and growth factors, among other proteins [18,19].

Unstimulated saliva production is secreted mostly by the submandibular glands. The submandibular glands’ saliva differs from the parotid glands—it is more viscous and mucin-rich [19]. The submandibular glands of PD patients also have the highest prevalence of Lewy-type synucleinopathy, ranging from 74% to 100%, depending on the tissue collection method [20,21,22,23]. This could provide a rationale for the preference of unstimulated saliva over the stimulated, watery saliva produced by parotid glands in the search for PD biomarkers.

LC-MS/MS mass spectrometry is a useful tool for biomarker candidate searches as it can identify large numbers of proteins across a large dynamic range and results in hypothesis-free analysis driven by the obtained data. In this study, we hypothesized that the involvement of salivary glands in synucleinopathy, as well as aberrations in protein secretion, may be reflected by changes in the saliva proteome of PD patients. Such changes in the composition of saliva could then be developed into a biomarker. The proteomic approach to saliva was initially introduced in studies of dental and oral diseases and is now applied in generalized conditions such as oncological disorders, addictions, or pediatric disorders [24,25,26,27]. The aim of this study is to compare the proteome of PD saliva vs. healthy controls (HC), as well as to further analyze specific pathways leading to different expression of proteins.

## 2. Materials and Methods

### 2.1. Study Group Characteristics and Material Collection

A total of 50 subjects were recruited for the study. Eleven out of fifty patients (9 controls and 2 in the PD group) were unable to provide enough saliva during the collection procedure.

The remaining 39 subjects recruited for the study were divided into two groups: 24 PD patients and 15 HC. All patients in the PD group were diagnosed in accordance with UK Brain Bank criteria for Parkinson’s disease. Detailed characteristics of the study groups are presented in Table 1. The mean duration of the disease in the PD group was 8.2 +/− 4.3 years. In total, 7 patients in the PD group were treated with a dopamine agonist (5-ropinirole, 5 piribedil, 1-rotigotine) in addition to levodopa treatment.

The study was approved by the Ethics Committee of the Medical University of Warsaw (KB/239/2015, with amendment KB/25/A/2016), and therefore was performed in accordance with the ethical standards laid down in the 1964 Declaration of Helsinki and its later amendments. All participants signed informed consent forms prior to their inclusion in the study. Participants were recruited between April 2018 and February 2019. Patients were recruited in the Department of Neurology, Faculty of Health Sciences.

Exclusion criteria included cigarette smoking, previous injection of botulinum toxin to salivary glands, treatment with anticholinergic medications, clinical diagnosis of neurodegenerative diseases other than PD, and known malignancies. Among PD patients, only levodopa and oral dopamine agonist treatments were allowed. HC were subjects with no clinical evidence of neurodegeneration, matched by age and sex to the PD group. The control group was screened and confirmed negative for symptoms of bradykinesia, tremor, imbalance, or rigidity, as well as REM behavior disorder history. The control group was recruited among invited individuals (site personnel and non-blood-related members of patients’ families or caregivers). No patients who had a periodontist, active inflammation, or oral cancer history were included.

Subjects were asked to refrain from drinking, eating, or using oral hygiene procedures for at least 2 h before the procedure and to rinse their mouths with tap water 30–60 min prior to collection. Saliva samples were collected in the morning hours, using RNA-Pro-Sal kits. The collection procedure with this device was described in detail by Chiang et al. [28]. Samples were immediately frozen at −80 °C after collection and later processed in the Mass Spectrometry Laboratory at the Institute of Biochemistry and Biophysics PAS.

### 2.2. Analytical Methods

#### 2.2.1. Sample Preparation for Mass Spectrometry Analysis

Mass spectrometry experiments were performed at the Mass Spectrometry Laboratory at the Institute of Biochemistry and Biophysics PAS. Freshly prepared urea buffer (9 M urea, 200 mM Tris-HCl pH 8.0 in MS-grade water) was aliquoted into 85 μL volumes and fully dried in a SpeedVac. The urea was redissolved in 50 μL of saliva to obtain 85 μL of protein sample in urea buffer. The protein concentration was measured with a BCA Protein Assay Kit (Thermo). Protein eluates were processed using single-pot, solid-phase-enhanced sample preparation (SP3) with some modifications (ultrasensitive proteome analysis using paramagnetic bead technology). Then, 40 μg of protein from each sample was transferred to a new tube and filled up to 100 μL with urea buffer. Cysteine bridges were reduced by 1 h incubation with 20 mM tris(2-carboxyethyl) phosphine (TCEP) at 37 °C followed by 30 min incubation at room temperature with 40 mM iodoacetamide (IAA). A magnetic bead mix was prepared by combining equal parts of Sera-Mag Carboxyl hydrophilic and hydrophobic particles (09-981-121 and 09-981-123, GE Healthcare). The bead mix was washed three times with MS-grade water and resuspended in a working concentration of 10 μg/μL. Then, 250 μg of the prepared bead mix, along with 5 μL of 10% formic acid and 800 μL of acetonitrile, were added to each sample. Proteins bound to beads were washed with 75% ethanol, isopropanol, and acetonitrile, followed by overnight digestion with 2 μg of trypsin/Lys-C mix (Promega). After digestion, peptides were washed with acetonitrile and eluted from the beads by subsequent incubation with MS-grade water and 2% DMSO with sonication during each step. Pulled aliquots were dried in a SpeedVac and resuspended in 40 μL 2% acetonitrile and 0.1% formic acid.

#### 2.2.2. Mass Spectrometry

Here, 2 ug of each saliva sample was analyzed using a nanoAcquity UPLC (Waters) directly coupled to a QExactive mass spectrometer (Thermo Scientific, Bremen, Germany). Peptides were trapped on a C18 precolumn (180 µm × 20 mm, Waters) with 0.1% FA in water as a mobile phase and transferred to a nanoAcquity BEH C18 column (75 µm × 250 mm, 1.7 µm, Waters) using ACN gradient (0–35% ACN in 160 min) in the presence of 0.1% FA at a flow rate of 250 nL/min. Measurements were performed in data-dependent mode with top 12 precursors selected for MS2. Full MS scans covering the range of 300–1650 *m*/*z* were acquired at a resolution of 70,000, with a maximum injection time of 60 ms and an AGC target value of 1e6. MS2 scans were acquired at a resolution of 17,500 and an AGC target value of 5e5. Dynamic exclusion was set to 30 s.

#### 2.2.3. Data Analysis

Obtained data were pre-processed with Mascot Distiller software (Matrixscience) and protein identification was performed using a Mascot Server 2.5 (Matrixscience) against the Homo sapiens protein sequences (20,490 sequences) deposited in the Swiss-Prot database (201,903, 559,634 sequences; 201,129,965 residues). The parameters were set as follows: enzyme—Trypsin; missed cleavages—2; fixed modifications—carbamidomethyl (C); variable modifications—oxidation (M); instrument—HCD. To reduce mass errors, peptide and fragment mass tolerance settings were established separately for each file after an off-line mass recalibration [29]. The assessment of confidence was based on a target–decoy database search strategy, as described by Elias et al., which provided q-value estimates for each peptide spectrum match [30,31]. All queries with q-values > 0.01, subset proteins, and proteins identified with one peptide were discarded from further analysis. The mass recalibration, FDR computations, and data filtering were done with Mscan software, developed in-house [32].

#### 2.2.4. Quantitative MS Data Processing

The lists of identified peptides were merged into one common list and overlayed onto 2-D heatmaps generated from LC-MS spectra and the volumes were obtained from the assigned peaks (a more detailed description of data extraction procedures can be found in [33]). The abundance of each peptide was determined as the height of a 2-D fit to the monoisotopic peak of the tagged isotopic envelope. Quantitative values were then exported into text files for statistical analysis with Diffprot software for non-parametric statistical analysis of differential proteomics data (Malinowska et al., 2012). Diffprot is an in-house software for statistical significance assessment. In this program, the statistical validity of the regulation or expression status of a protein represented by its calculated protein ratio is based solely on the statistical analysis of the datasets from a given experiment, without assumptions on the character of the distribution of peptide ratios in a dataset (e.g., its normality). The probability of obtaining a given protein ratio by random selection from the dataset was tested by calculating protein ratios for a large number of permuted decoy datasets in which the peptide–protein assignment was scrambled. Unfortunately, calculated *p-*values in this dataset were non-significant for identified proteins, so it was decided to report only raw *p*-values. Diffprot was run with the following parameters: number of random peptide sets = 10^6^; clustering of peptide sets—only when 90% identical; normalization by LOWESS, min-pep 4, quantification based on unique peptides. Only proteins with fold changes >1.5 were taken into consideration during further analysis.

The ROC plots were obtained by plotting all sensitivity values (true positive rate— TPR) on the y-axis against their equivalent (1-specificity) values (false positive rate—FPR) for all available thresholds on the x-axis using ROCit 2.1.1 software (authors: R.A Khan, T. Brandenburger). The area under the curve (AUC) was calculated to provide a summary of overall potential marker effectiveness. An optimal point was chosen as described by Youden [34].

Proteins with at least 1.5-fold change between groups were analyzed using Ingenuity Pathway Analysis (IPA; QIAGEN, Germany) software to identify relevant biological pathways and upstream regulators.

## 3. Results

**Figure 1:**
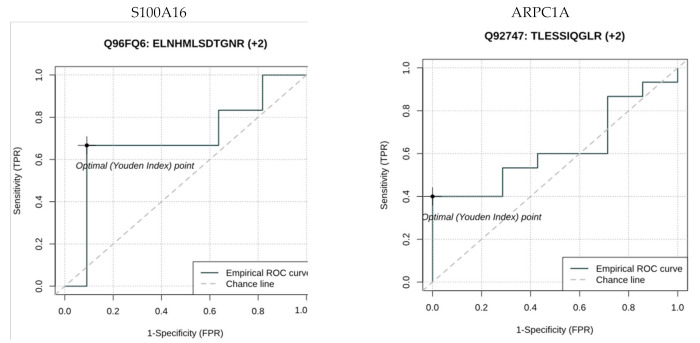
Area under the curve (AUC) receiver operating characteristic curve (ROC) analysis for S100A16 and ARPC1A proteins in HC versus PD patients.

A total of 1328 peptides corresponding to 530 proteins were identified. We observed a −10.47-fold change in the concentration of protein S100-A16 in the PD group vs. healthy control. We also observed changes in concentrations of proteins from the annexin family (annexin A2 (−4.4-fold change) and annexin A8 (−3.84)) in PD vs. control. The resistin concentration was 4.04 times lower in PD than in control. The proteins with the highest and lowest fold changes for MSP/HC peak areas (>1.5/<−1.5) are presented in Supplementary Table S1. Protein S100-A16, actin-related protein 2/3 complex subunit 1A (ARPC1A), and vacuolar protein sorting-associated protein 4B (VPS4B) had *p*-values < 0.05 in Diffprot software analysis [29]. Unfortunately, we observed heterogenous expression of salivary proteins among samples, with the mean percentage of proteins detected in each sample at around 32%. In effect, the data did not achieve enough statistical power to give significant results after FDR. Therefore, in Supplementary Table S1, we show raw *p*-values for proteins with a fold change >1.5 or <−1.5 in PD versus HC saliva samples. Figure 1 represents the results of AUC ROC calculated for 2 proteins selected based on high fold changes and low *p*-values—S100A16 and ARPC1A. The values were AUC = 0.7, specificity = 0.67, sensitivity = 0.91 for S100A16; AUC = 0.62, specificity = 1, sensitivity = 0.4 for ARPC1A. The third interesting protein with a good *p*-value, VPS4B, was characterized by lower AUC ROC of 0.54, with 100% sensitivity and specificity of 0.4.

**Figure 2:**
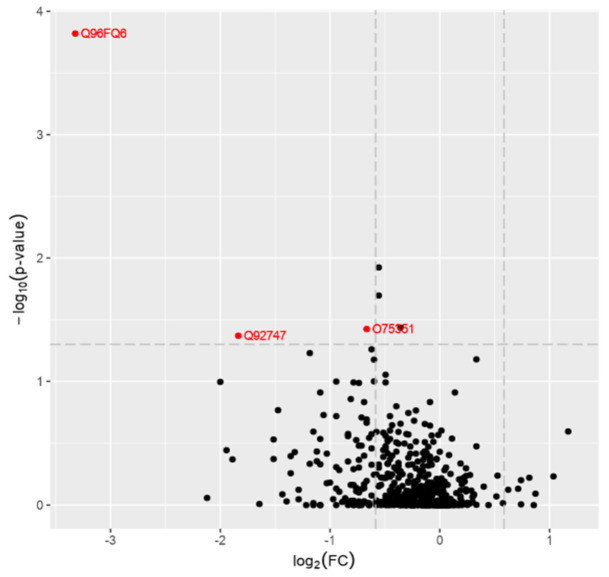
A volcano plot from the mass spectrometry data is shown. The x-axis shows the fold change values and the y axis shows the −log_10_ p-values showing statistical significance. Q96FQ6–S100A16 protein; Q92747–ARPC1A protein; O75351–VPS4B protein. The horizontal dashed line shows where *p* = 0.05 (−log_10_(0.05)~ = 1.3) is, while the vertical dashed line shows where the fold change is 1.5 (log_2_ (1.5)~ = 0.58). The absolute 1.5-fold change and *p*-value of 0.05 are used as the threshold cutoff.

To visualize the proteomic findings in samples, we provide a volcano plot reporting *p-*values against fold changes (Figure 2).

Next, we investigated networks of proteins with fold changes above 1.5 using IPA to further explore molecular processes possibly leading to observed changes. We performed upstream regulator analysis to predict molecules that may have been causing observed protein expression changes. Table 2 contains a list of upstream regulators identified with the highest and lowest predicted z-scores.

## 4. Discussion

To the best of our knowledge, this is one of the very first studies to assess the salivary proteomes of PD patients and healthy subjects. The results of our analysis, as well as previous studies on PD saliva, indicate that the salivary proteome of PD patients might differ from that of healthy controls. A study by Kumari et al. took a metabolomic approach to the assessment of saliva from PD patients. The authors described increased concentrations of N-acetylglutamate, acetoin, acetate, alanine, phenylalanine, tyrosine, histidine, glycine, acetoacetate, taurine, TMAO, GABA, fucose, propionate, isoleucine, and valine in PD patients versus healthy controls (HC). They also indicated that subgroup analysis revealed differences in metabolite concentrations in saliva among different stages of the disease [35]. Masters et al. reported preliminary results from a study involving 3 PD patients and one control. They reported upregulation of S100-A9 and S100-A8 proteins in the saliva of PD patients versus the control [36]. In our larger cohort, we observed lower concentrations of the S100A16 protein in the PD group vs. control. It also had the highest AUC of all the identified proteins. Higher detection of the S100B family of proteins is associated with numerous neurodegenerative processes, including Alzheimer’s disease, PD, and amyotrophic lateral sclerosis [37]. It was also proposed as a biomarker of neural injury and clinical outcome in stroke and trauma [38,39]. S100B protein overexpression has an established role in PD. It was associated with age of onset of PD and neuronal and glial damage [40,41]. To our best knowledge, no studies on salivary S100A16 protein measurements have been performed. S100A16 participates in calcium ion binding and adipocyte differentiation. In clinical settings, its role has mainly been investigated in oncogenesis. Specifically, S100A16 expression was upregulated in tumors of the lungs, thyroid gland, pancreas, bladder, and ovaries [42]. Active neoplastic process was, however, an exclusion criterion in our cohort. The S100A16 protein’s higher expression was also described as promoting adipocyte differentiation in animal models [43,44]. We can only speculate that lower salivary levels of this protein can reflect malnutrition and lower adipose tissue levels in the PD group [45,46].

ARP2/3 participates in polymerization of actin upon stimulation via nucleation-promoting factor. The Arp2/3 complex takes part in the formation of branched actin networks in the cytoplasm. This process provides the force for cell motility. The Arp2/3 complex is also involved in homologous recombination repair after DNA damage [47]. To our best knowledge, changes of expression of this protein were not associated previously with neurodegeneration. We observed lower expression of ARP2/3 in PD vs. HC. Animal studies prove that accumulation of alpha-synuclein can induce DNA single-strand and double-strand breaks [48,49], which are not limited to the central nervous system. We hypothesize that lower expression of the Arp2/3 complex may reflect an inefficient repair mechanism of DNA damage in the PD cohort.

VPS4B is involved in the endosomal multivesicular body pathway. It is required for the exosomal release of SDCBP, CD63, and syndecan [50]. In our cohort, lower expression of VPS4B in saliva was observed in the PD group. Simultaneous inhibition of VSP4A and VSP4B reduces exosome secretion [50]. Exosomes play a crucial role in alpha-synuclein propagation [51,52]. A pilot study by Rani et al. indicated that PD patients have increased secretion of exosomes from neuronal endings in salivary glands [53]. A lower concentration of VPS4B could perhaps reflect its higher uptake in the exosome formation process in salivary glands. In our study, although VPS4B had a very high sensitivity of 100%, this classifier’s effectiveness is insufficient.

Although the proteomic data on PD are limited, there have been several genomic studies on this disease [54,55,56]. The GWAS study by Nalls et al. revealed 90 PD-related genetic risk loci [55]. In our proteomic study, we did not identify any proteins encoded by these genes. However, the study by Nalls et al. showed the *VPS13C* loci as a potential PD risk factor. The VPS13C protein, similarly to the VPS4B protein discovered in our study, is involved in the endosomal multivesicular bodies pathway. It also increases PINK1/Parkin-dependent mitophagy. Additionally, Lesage et al. identified VPS13C as a gene responsible for autosomal recessive PD [57].

Interestingly, some of the proteins generally associated with inflammation had lower concentrations in PD group than in control group. This proves that oral inflammation related to worse oral hygiene in PD is not the main factor determining differences in saliva composition between groups [58]. Regarding proteins with high fold change (>4) but no significant difference in concentration between cohorts, we identified proteins from the annexin family and resistin. Inhibition of NF-κ B pathways observed in analysis of upstream regulators may coincide with lower concentrations of salivary resistin. NF-κ B plays a pivotal role in PD-related neuroinflammation. Numerous substances working through inhibition of NF-κ B pathways were investigated regarding their neuroprotective role in PD models [59,60,61,62]. Its expression is also increased in the substantia nigra of PD patients [63]. Our results indicate that this process is not reflected by saliva composition.

The high number of identified upstream regulators with high z-scores may reflect the complex nature of the disease, which is partially reflected in the salivary composition. The molecules identified as possibly driving differential expression of proteins between PD and HC groups were mainly cytokines, transcription regulators, chemicals, and kinase inhibitors. We observed an activation of an upstream regulator previously associated with PD pathogenesis: PD98059. PD98059 is an ERK1/2 signaling inhibitor with implications for antidyskinetic effects in PD [64,65].

Inhibition of prolactin-related proteins may be in line with previously reported effects of levodopa treatment on prolactin secretion [66,67,68].

We did not detect alpha-synuclein in our samples, which may be due to method limitations. Recent findings did not confirm that salivary alpha-synuclein can differentiate between HC and PD patients [4].

A limitation of our study was the high variability of salivary protein expression, with only around 30% of the total number of proteins identified in each sample. The saliva proteome may be significantly affected by such general aspects as patients’ oral cavity status, type of diet, nutrition, and comorbidities. Treatment received for PD (levodopa, domperidone, botulinum toxin) may also influence saliva production and composition [69,70]. These variables led to vast changes in the results observed among our subjects. We took measures to unify collection procedures and exclude some possibly interfering factors. Our goal was, however, to include heterogenous patients with PD without artificially narrowing study groups and to reflect the real-life proteomic profile of saliva. The findings of this study should be validated independently with Western blotting or enzyme-linked immunosorbent assay to increase its accuracy.

It remains of interest whether changes in the salivary proteome may appear ahead of motor symptoms of PD. In particular, markers of inflammation could serve as its early indicators. We can only hypothesize that this may be the case, since gastroenteric symptoms appear in PD years before motor manifestation. Salivary glands and gastroenteric plexuses are positive for Lewy pathology, with similar high frequency [21]. This “liquid biopsy” could then result in early causative treatment.

This study’s focus on saliva is both its limitation and advantage. Our study reveals several interesting directions for future studies of the salivary proteome in PD. It suggests that inflammatory processes and imbalances in cellular pathways associated with PD pathology may be reflected in the composition of saliva. The differences observed between the salivary proteomic profiles of PD patients and HCs add to the current knowledge about the state of the widespread pathology underlying the disease. However, very little is known about salivary changes in neurodegeneration; therefore, some findings are difficult to interpret. Further studies will have to be conducted to evaluate the levels of selected salivary proteins at different stages of PD. A more detailed assessment of received therapies, dental status, and motor subtype of PD may all also play a role in the proteomic profile of saliva. We believe that a proteomic and bioinformatic approach highlights the potential of salivary diagnostics in understanding the pathophysiology of PD and opens the way for future biomarker research.

## Figures and Tables

**Table 1:** Clinical characteristic of study groups. LED: levodopa equivalent dose; UPDRS: Unified Parkinson’s Disease Rating Scale; ns: non- significant; na: non applicable.

	PD Group	Healthy Control	*p*-Value
Number of patients	24	15	
Sex M/F	15/9	9/5	ns
Age	61.6 ± 8.2	60.9 ± 6.7	ns
Levodopa dose	1352 ± 763	na	
LED	1524 ± 786	na	
UPDRS part III OFF	34.6 ± 12.4	na	

**Table 2:** Upstream regulators identified using information from the Ingenuity Pathway Analysis (IPA) software (Qiagen, Germany).

Upstream Regulator	Molecule Type	Predicted Activation State	Activation z-Score	*p*-Value of Overlap	Target Molecules in Dataset
PD98059	chemical - kinase inhibitor	Activated	2.737	9.13 × 10^−5^	ANXA1⇓,CAMP⇓, FN1⇓, GRN⇓,ITGAM⇓, KRT19⇓, NQO1⇓, RETN⇓, S100A4⇓, S100A8⇓,SOD1⇓
SRF	transcription regulator	Activated	2.646	2.72 × 10^−4^	CAMP⇓, ELANE⇓, ITGAM⇓, LTF⇓, MYH9⇓, S100A8⇓, S100A9⇓, SLPI⇓
SB203580	chemical - kinase inhibitor	Activated	2.596	8.58 × 10^−5^	ANXA1⇓, ANXA3⇓, CAMP⇓, FN1⇓, HMGB1⇑, ITGAM⇓, NQO1⇓, RETN⇓, SLPI⇓, TGM1⇓
RICTOR	other	Activated	2.236	1.11 × 10^−2^	PSMA1⇓, PSMA5⇓, PSMB1⇓, PSMB3⇓, RPSA⇓
GATA3	transcription regulator	Activated	2.216	1.22 × 10^−3^	FN1⇓, PPL⇓, S100A8⇓, S100A9⇓, SLPI⇓, TGM1⇓
mifepristone	chemical drug	Activated	2.121	1.75 × 10^−4^	ANXA1⇓, CAST⇓,GSTM1⇓, HSPD1⇓, ITGAM⇓, JUP⇓, LTF⇓, PSMA1⇓
tert-butyl-hydroquinone	chemical reagent	Inhibited	−2.175	6.90 × 10^−5^	GSS⇓, ITGAM⇓, ME1⇓, NQO1⇓, PSMA5⇓
arsenic trioxide	chemical drug	Inhibited	−2.176	1.08 × 10^−3^	FN1⇓, ITGAM⇓, ME1⇓, NQO1⇓, PDIA4⇓, S100A8⇓, VCP⇓
IL17A	cytokine	Inhibited	−2.196	8.72 × 10^−3^	CAMP⇓, MPO⇓, S100A12⇓, S100A8⇓, S100A9⇓
SP1	transcription regulator	Inhibited	−2.200	1.91 × 10^−5^	CAMP⇓, CES1⇓, ELANE⇓, FN1⇓, GSS⇓, ITGAM⇓, KRT16⇓, KRT19⇓, KRT4⇓, PADI4⇑, SOD1⇓, TGM1⇓
IL2	cytokine	Inhibited	−2.213	3.35 × 10^−2^	ANXA1⇓,FCGR3A/FCGR3B⇑,GRN⇓, HSPD1⇓, PSMB1⇓, S100A4⇓, S100A8⇓
butyric acid	chemical - endogenous mammalian	Inhibited	−2.227	2.81 × 10^−8^	ALDH1A1⇓, ANXA1⇓, ANXA5⇓, CAMP⇓, CEACAM5⇓, ELANE⇓, GRN⇓, HMGB1⇑, ITGAM⇓, KRT13⇓, MVP⇓, PCMT1⇑, TGM1⇓, TP53I3⇓
curcumin	chemical drug	Inhibited	−2.236	7.96 × 10^−3^	CAMP⇓, HMGB1⇑, ITGAM⇓, NQO1⇓, RETN⇓, SOD1⇓
OSM	cytokine	Inhibited	−2.272	5.08 × 10^−7^	ANXA1⇓, ANXA3⇓, CAMP⇓, CDA⇓, FN1⇓, GCA⇓, KLK13⇑, KRT16⇓, KRT19⇓, LRRFIP1⇓, S100A12⇓, S100A8⇓, S100A9⇓, SLPI⇓
HSF1	transcription regulator	Inhibited	−2.360	6.56 × 10^−4^	CCT2⇓, CCT3⇑, FKBP4⇓, HMGB1⇑, HSPD1⇓, TCP1⇓
1,2-dithiol-3-thione	chemical reagent	Inhibited	−2.503	6.27 × 10^−8^	CCT3⇑, GSTM1⇓, NQO1⇓, PDIA4⇓, PSMA1⇓, PSMA5⇓, PSMB1⇓, PSMB3⇓, SOD1⇓, VCP⇓
EGF	growth factor	Inhibited	−2.516	1.16 × 10^−5^	FN1⇓, ITGAM⇓, KRT16⇓, KRT19⇓, KRT5⇓, LTF⇓, PFKM⇓, PSMB1⇓, PSMB3⇓, S100A4⇓, S100A9⇓, TGM1⇓
tetradecanoylphorbol acetate	chemical drug	Inhibited	−2.608	1.43 × 10^−5^	ANXA1⇓, AZU1⇓, CES1⇓, ELANE⇓, GSTM1⇓, ITGAM⇓, LRRFIP1⇓, MPO⇓, MYH9⇓, NQO1⇓, RETN⇓, S100A14⇓, S100A8⇓, S100A9⇓, SLPI⇓, SOD1⇓, STATH⇑, TGM1⇓
NFkB (complex)	complex	Inhibited	−2.621	3.90 × 10^−3^	ALDH7A1⇓, CAMP⇓, CAPNS1⇓, FN1⇓, HSPA9⇓, ITGAM⇓, KRT19⇓, PKP1⇓, SLPI⇓
KLF4	transcription regulator	Inhibited	−2.704	2.55 × 10^−7^	ALDH1A1⇓, DSP⇓, FN1⇓, ITGAM⇓, KRT13⇓, KRT19⇓, PFKP⇓, PPL⇓, S100A14⇓, SLPI⇓, TGM1⇓
CEBPA	transcription regulator	Inhibited	−2.755	1.11 × 10^−5^	ANXA1⇓, CAMP⇓, ELANE⇓, ITGAM⇓, LTF⇓, MPO⇓, PPL⇓, RETN⇓, S100A8⇓, S100A9⇓, SOD1⇓
Lipopolysaccharide	chemical drug	Inhibited	−2.951	9.57 × 10^−9^	ANXA1⇓, ANXA3⇓, ANXA5⇓, AZU1⇓, CAMP⇓, CLIC3⇓, ELANE⇓, FN1⇓, GCA⇓, HMGB1⇑, ITGAM⇓, ITIH4⇓, KRT13⇓, KRT4⇓, LGALS3⇓, LRRFIP1⇓, LTF⇓, MPO⇓, MYH9⇓, NQO1⇓, ORM2⇓, PDIA4⇓, PFKP⇓, RETN⇓, S100A12⇓, S100A8⇓, S100A9⇓, SLPI⇓, SOD1⇓, TRIM29⇓
PRL	cytokine	Inhibited	−3.071	1.07 × 10^−6^	ANXA3⇓, ANXA5⇓, FN1⇓, GSTM1⇓, HSPD1⇓, KRT19⇓, KRT5⇓, PDIA4⇓, RPSA⇓, SOD1⇓
NFE2L2	transcription regulator	Inhibited	−3.263	2.58 × 10^−8^	CCT3⇑, FN1⇓, GSS⇓, GSTM1⇓, HSPA9⇓, ME1⇓, NQO1⇓, PDIA4⇓, PSMA1⇓, PSMA5⇓, PSMB1⇓, PSMB3⇓, SOD1⇓, VCP⇓
beta-estradiol	chemical - endogenous mammalian	Inhibited	−3.428	6.34 × 10^−8^	ADK ⇓,ALDH7A1⇓, ANXA1⇓, ANXA3⇓, CAST⇓,CCT2⇓, DSP⇓, FN1⇓, HNRNPD⇓, HSPA9⇓, HSPD1⇓, ITIH4⇓, KRT13⇓, KRT16⇓, KRT19⇓, KRT4⇓, KRT5⇓, LGALS3⇓, LTF⇓, MPO⇓, PADI4⇑, PDIA4⇓, PPL⇓, PSMA1⇓, PSMB1⇓, S100A4⇓, S100A9⇓, SLPI⇓, TP53I3⇓, TRIM29⇓

Knowledge base to explain observed proteomic changes between the patients and the control group. The table contains regulators with the highest and lowest Z-score (cut-off at > 2-activated or < −2-inhibited) identified from the IPA software (Qiagen, Germany) with p-values overlapping < 0.05. Targets in the experimental results are labeled as ⇓ for downregulated or ⇑ for upregulated proteins between the patient and the control group. The colors of the target genes indicate the literature prediction of the influence of the upstream regulator on the given protein: red—activated; blue—inhibited; black—affected with no proven direction of the relationship.

## Data Availability

The data supporting this study are available in PRIDE using accession number PXD023489.

## Supplementary Materials

The following are available online at https://www.mdpi.com/article/10.3390/brainsci11050661/s1; Table S1: The protein clusters with fold changes >1.5 or <−1.5 in Parkinson patients (PD) versus healthy control (HC) saliva samples.
