# Supplementary material for: Proteomic Profile of Saliva in Parkinson’s Disease Patients: A Proof of Concept Study
Source: Brain Sci. 2021 May 18;11(5):661. doi: 10.3390/brainsci11050661 (PMC8158489; doi:10.3390/brainsci11050661)
Supplement: Supplementary file 1 [file brainsci-11-00661-s001.zip › brainsci-1198346-supplementary.pdf]

Supplementary

Table S1. The protein clusters with fold changes >1.5 or <-1.5 in Parkinson patients (PD) versus healthy control (HC) saliva samples. Proteins with significantly different expression between PD and HC patients are bolded. Orange proteins have higher expression in PD group than HC group. Blue proteins have lower expression in PD group than HC group.

Protein	*p-value*	*fold change*	*Description*
**Q96FQ6**	**0.0002**	**-10.47**	**Protein S100-A16**
A6NMY6 + P07355	0.875	-4.4	Putative annexin A2-like (Annexin A2)
Q9HD89	0.101	-4.04	Resistin
Q5VT79 + P13928	0.36	-3.84	Annexin A8-like protein 1<br><br>Annexin A8
Q9BYZ2	0.427	-3.72	L-lactate dehydrogenase A-like 6B
**Q92747**	**0.043**	**-3.57**	**Actin-related protein 2/3 complex subunit 1A**
Q8N1N4	0.979	-3.15	Keratin, type II cytoskeletal 78
Q9BTM1+ P0C0S8+ Q7L7L0+ P20671+ Q99878+ Q16777+ Q93077+ Q96KK5+ Q6FI13+ P04908+ O75367+ Q8IUE6+ P16104	0.295	-2.83	Histone H2A subtypes
P14923 + P35222	0.424	-2.83	Junction plakoglobin
P49419	0.171	-2.81	Alpha-aminoadipic semialdehyde dehydrogenase
Q14764	0.818	-2.73	Major vault protein
P59665 + P59666	0.934	-2.64	Neutrophil defensin 1
P53675 + Q00610	0.553	-2.6	Clathrin heavy chain 1
P26447	0.402	-2.57	Protein S100-A4
P15559	0.373	-2.53	NAD(P)H dehydrogenase [quinone] 1
P20160	0.752	-2.46	Azurocidin
P04196	0.897	-2.43	Histidine-rich glycoprotein
P04083	1	-2.31	Annexin A1
Q13835	0.464	-2.28	Plakophilin-1
P02751	0.059	-2.27	Fibronectin
Q9UM07	0.254	2.25	Protein-arginine deiminase type-4
P28066	0.255	-2.23	Proteasome subunit alpha type-5
P48668+ P04259+ O95678+ P02538	1	-2.21	Keratin, type II cytoskeletal 6C, 6B 6A
P15924	0.964	-2.21	Desmoplakin
Q86VP6	0.369	-2.16	Cullin-associated NEDD8-dissociated protein 1

Q9HCY8	0.442	-2.16	Protein S100-A14
Q13421	0.292	-2.14	Mesothelin
P19013	1	-2.13	Keratin, type II cytoskeletal 4
P22735	0.468	-2.13	Protein-glutamine gamma-glutamyltransferase K
P50995	0.123	-2.12	Annexin A11
P13646	1	-2.12	Keratin, type I cytoskeletal 13
P06731	0.187	-2.1	Carcinoembryonic antigen-related cell adhesion molecule 5
P09488 + Q03013	0.384	-2.06	Glutathione S-transferase Mu 1
P22061	0.586	2.05	Protein-L-isoaspartate(D-aspartate) O-methyltransferase
Q9H1E3	0.665	-2.04	Nuclear ubiquitous casein and cyclin-dependent kinase substrate 1
P08865	0.658	-2	40S ribosomal protein SA
P10809	0.896	-1.97	60 kDa heat shock protein, mitochondrial
P48637	0.493	-1.94	Glutathione synthetase
P13647 + Q5XKE5	1	-1.93	Keratin, type II cytoskeletal 5,<br>Keratin, type II cytoskeletal 79
P38646	0.100	-1.93	Stress-70 protein, mitochondrial
Q14103	0.191	-1.92	Heterogeneous nuclear ribonucleoprotein D0
P62136 + P62140 + P36873	0.735	-1.92	Serine/threonine-protein phosphatase PP1-alpha, beta and gamma catalytic subunits
P08133	0.521	-1.9	Annexin A6
P80511	0.78	-1.9	Protein S100-A12
P49913	0.944	-1.84	Cathelicidin antimicrobial peptide
P49368	0.809	1.83	T-complex protein 1 subunit gamma
P11216	0.879	-1.82	Glycogen phosphorylase, brain form
P02808	1	1.81	Statherin
Q14624	0.277	-1.79	Inter-alpha-trypsin inhibitor heavy chain H4
O95833	0.444	-1.79	Chloride intracellular channel protein 3
P48163	0.266	-1.78	NADP-dependent malic enzyme
P12429	0.956	-1.78	Annexin A3
P05109	0.999	-1.77	Protein S100-A8
P20810	0.922	-1.77	Calpastatin
P09429 + B2RPK0	0.601	1.76	High mobility group protein B1<br>Putative high mobility group protein B1-like 1
Q5D862	0.139	-1.75	Filaggrin-2
P23141	0.687	-1.75	Liver carboxylesterase 1
P03973	0.997	-1.74	Antileukoproteinase
P00352 + P51648	0.688	-1.73	Retinal dehydrogenase 1
P00441	0.102	-1.72	Superoxide dismutase [Cu-Zn]
P08779	0.512	-1.71	Keratin, type I cytoskeletal 16

P08727	0.949	-1.71	Keratin, type I cytoskeletal 19
P06702	1	-1.71	Protein S100-A9
P25786	0.921	-1.71	Proteasome subunit alpha type-1
Q14134	0.726	-1.7	Tripartite motif-containing protein 29
P80723	0.987	-1.7	Brain acid soluble protein 1
Q9HC38	0.298	-1.7	Glyoxalase domain-containing protein 4
O00748	0.772	-1.68	Cocaine esterase
Q6P4A8	0.791	-1.68	Phospholipase B-like 1
P02749	0.103	-1.68	Beta-2-glycoprotein 1
P10163	1	-1.67	Basic salivary proline-rich protein 4
Q9UKR3	0.986	1.67	Kallikrein-13
P13667	0.497	-1.67	Protein disulfide-isomerase A4
P11215	0.431	-1.67	Integrin alpha-M
Q08554	0.629	1.67	Desmocollin-1
Q09666	1	-1.67	Neuroblast differentiation-associated protein AHNAK
P17987	0.331	-1.67	T-complex protein 1 subunit alpha
P55072	0.933	-1.65	Transitional endoplasmic reticulum ATPase
P28799	0.331	-1.65	Progranulin
P20618	0.196	-1.65	Proteasome subunit beta type-1
P08246	0.743	-1.65	Neutrophil elastase
P05164	1	-1.64	Myeloperoxidase
P51570	0.738	1.64	Galactokinase
Q01813	0.678	-1.62	ATP-dependent 6-phosphofructokinase, platelet type
Q53FA7	0.399	-1.62	Quinone oxidoreductase PIG3
P08758	0.147	-1.61	Annexin A5
Q9H361+ Q4VXU2 + P11940+ Q13310	0.730	-1.61	Polyadenylate-binding protein 3.1-like,1 and 4
P17931	0.931	-1.6	Galectin-3
P55263	0.216	-1.59	Adenosine kinase
Q8TE68	0.203	-1.59	Epidermal growth factor receptor kinase substrate 8-like protein 1
**O75351**	**0.038**	**-1.59**	**Vacuolar protein sorting-associated protein 4B**
Q99877+ P06899+ Q5QNW6+ P23527+ Q99879+ Q16778+ P62807+ Q8N257+ P33778+ O60814+ P57053+ Q99880+ Q93079+ P58876	0.958	-1.59	Histone H2B subtypes
P28676	0.638	-1.58	Grancalcin
P02788	1	-1.57	Lactotransferrin

P35579	1	-1.56	Myosin-9
P78371	0.286	-1.56	T-complex protein 1 subunit beta
O60437	0.964	-1.56	Periplakin
P27824	0.267	-1.54	Calnexin
P32320	0.055	-1.54	Cytidine deaminase
O75015 + P08637	0.752	1.54	Low affinity immunoglobulin gamma Fc region receptor III-A
A0A0C4DH42	0.837	-1.54	Immunoglobulin heavy variable 3-66
Q32MZ4	0.348	-1.53	Leucine-rich repeat flightless-interacting protein 1
P08237 + P17858	0.472	-1.53	ATP-dependent 6-phosphofructokinase, liver type
P49720	0.568	-1.53	Proteasome subunit beta type-3
P04632	0.067	-1.52	Calpain small subunit 1
Q02790	0.100	-1.51	Peptidyl-prolyl cis-trans isomerase FKBP4
P19652	0.565	-1.5	Alpha-1-acid glycoprotein 2
Q08188	1	-1.5	Protein-glutamine gamma-glutamyltransferase E
Q13126	0.917	-1.5	S-methyl-5’-thioadenosine phosphorylase
